# Assessing the impact of the COVID-19 pandemic on small and medium-sized enterprises performance

**DOI:** 10.3389/fpsyg.2022.927628

**Published:** 2022-10-11

**Authors:** Aries Susanty, Nia Budi Puspitasari, Arfan Bakhtiar, Feby Prasetya

**Affiliations:** Industrial Engineering Department, Diponegoro University, Semarang, Indonesia

**Keywords:** COVID-19 pandemic, multi-attribute value theory approach, K-means cluster analysis, discriminant analysis, SMEs, vulnerability

## Abstract

This study has several purposes. First, identify indicators contributing to the performance of small and medium-sized enterprises (SMEs) that could be affected by the COVID-19. Second, formulate the framework to measure the level of vulnerability of SMEs. Third, assign the SMEs into several clusters. Data used in this research were collected through web-based closed questionnaires and short telephone interviews. This study used Content Validity Analysis, Analytical Hierarchy Process, Multi-Attribute Value Theory approach, *K*-means Clustering Analysis, and Discriminant Analysis for data processing. The data processing results indicated that the 44 valid indicators belonging to ten dimensions could be used to measure the level of vulnerability of SMEs whose performance was affected by the COVID-19 pandemic. The surveyed SMEs can be segmented into four clusters, namely resilient cluster, low vulnerability cluster, moderate vulnerability cluster, and high vulnerability cluster. Most of the surveyed SMEs belong to the moderate and high vulnerability clusters. The differences between the clusters were based on 16 indicators. These indicators include levels of supplier disruption and the SMEs’ market in which the SMEs operate or expect to operate. The results of this study help quantify how the pandemic could generate different levels of impact on each indicator that could depend on the business and what policymakers should consider as they contemplate the scale of the required intervention. Overall, this study contributes to the literature on the effects of the pandemic on SMEs by synthesizing the findings of studies on the impact of COVID-19 on SMEs. The study also determined the framework and the equation for measuring the level of SME vulnerability caused by the pandemic.

## Introduction

The negative impact of the COVID-19 pandemic has been recorded in every aspect of life, with economic, political, social, and psychological implications (Bretas and Alon, 2020; Ratten, 2020). Although the main impact of this pandemic is on human health and human health perception (Akpan et al., 2020), Akbulaev et al. (2020) highlight the multi-faceted impact of the COVID-19 crisis. Besides human health and human health perception, the COVID-19 pandemic affects companies’ supply and demand and then forces them to operate in the new condition. During the pandemic, some industries have shown a certain level of resilience or even found a new operating niche and most small and medium-sized enterprises (SMEs) in several sectors found themselves in “new normal” operating environments (Gregurec et al., 2021). There is anxiety about how the pandemic and the government’s response to it (lockdowns, social distancing guidelines, etc.) will affect SMEs. This anxiety is significant since SMEs represent over 90% of all firms worldwide. This condition makes SMEs the backbone of the world economy in the formal and informal business sectors (Tannenbaum et al., 2020). SMEs constitute a significant part of the private sector in most developed and developing countries (Beck and Demirguc-Kunt, 2006). Despite their critical role, SMEs are the most threatened by the COVID-19 crisis, given their relatively vulnerable financial position (Ghosal and Ye, 2015; Doshi et al., 2018). Their vulnerability has triggered many government programs, including financial assistance, wage subsidies, and payment deferrals (OECD, 2020). Mainly, SMEs face many issues in managing their business, revenues, and finances (Shahbaz et al., 2020). The pandemic has significantly affected small companies due to their limited financial resources and business scale (Carruthers, 2020).

Given that Indonesian SMEs absorb approximately 97% of the total workforce in the economic sector, contributing about 61.41% to gross domestic product (GDP) in 2018 (Hidayati and Rachman, 2021), it is crucial to understand the negative impact of the COVID-19 pandemic on the GDP. Some researchers (such as Tairas, 2020; Saturwa et al., 2021) and government institutions present descriptive statistics explaining the impact of COVID-19 on Indonesian SMEs. Based on 1,332 complaints from SMEs in 18 provinces, the Indonesian Ministry of Cooperatives and Small and Medium Enterprises identified several issues facing SMEs during the COVID-19 pandemic. These issues include declining sales, difficulty obtaining raw materials, stagnant distribution caused by lockdown, difficulty securing capitalization, and low productivity caused by work hour restrictions (Kemenkop-UKM, 2020). About 917 SMEs (69%) experienced decreased sales turnover. Approximately 119 SMEs (9%) had difficulty distributing manufactured goods, and about 179 SMEs (13%) had difficulty accessing business capital. Moreover, approximately 50 SMEs (4%) experienced a drastic reduction in production that temporarily stopped production. Although the result of this study does not represent the overall conditions of SMEs in Indonesia, which consists of 59–62 million businesses unit, the results may indicate that SMEs in Indonesia experienced considerable pressure because of the pandemic.

Despite the attempts by several studies to explain the impact of the pandemic on Indonesian SME performance, it was not easy to find research that explicitly studied the significant indicators and framework for measuring the impact of the pandemic and then clustering the SMEs based on the level of the impacts. Accordingly, this study seeks to provide insights into the following research questions.

1. What indicators contribute to SME performance because of the COVID-19 pandemic?
2. How should the framework be formulated to measure SME vulnerability during the pandemic?
3. Should SMEs be assigned into clusters according to similar indicators reflecting the impact of the pandemic?

Based on these three research questions, our study contributes to the emerging body of literature on the performance management measurement of SMEs (such as Ng et al., 2020; Rojas-Lema et al., 2020; Sardi et al., 2020; Mendy, 2021; Ndzana et al., 2021) and the effects of the COVID-19 pandemic on SMEs (Al-Fadly, 2020; Bartik et al., 2020; Fabeil et al., 2020; Humphries et al., 2020; Lu et al., 2020; Lutfi et al., 2020; Omar et al., 2020; Ratnasingam et al., 2020; Shafi et al., 2020; Tairas, 2020; Dai et al., 2021; Nawaiseh, 2021; Saturwa et al., 2021). Basically, not only about the effect of the COVID-19 pandemic on the SMEs, the previous studies have been carried out on COVID-19 from different perspectives like digital learning during the emergence of COVID-19 virus (Hasan and Bao, 2020; Aditya, 2021; Chaturvedi et al., 2021; Deshpande and Mhatre, 2021; Smith et al., 2021), its impact on the economies of different countries (Ye et al., 2020; Ali et al., 2021; Bhattacharya and Banerjee, 2021; Cuschieri and Grech, 2021; Delbiso et al., 2021; Donnarumma and Pezzulo, 2021; Mahi et al., 2021; Prempeh, 2021; Roy et al., 2021), its role in the global health crisis (Ankrah et al., 2021; Chaturvedi et al., 2021; Chirisa et al., 2021; Donnarumma and Pezzulo, 2021; Hannam-Swain and Bailey, 2021; Prempeh, 2021; Sarfraz et al., 2021; Wang et al., 2021; Zhao and Zhou, 2021), the worst of all its impact on the mental wellbeing of people (Ciotti et al., 2020; Elmer et al., 2020; Filipova et al., 2020; Lee, 2020; Serafini et al., 2020; Adom et al., 2021; Chaturvedi et al., 2021; Coupet et al., 2021; Das and Bhattacharyya, 2021; Deshpande and Mhatre, 2021; Hannam-Swain and Bailey, 2021; Kareem, 2021; Li and Cao, 2021; Pandya and Lodha, 2021; Saha et al., 2021; Tonkin and Whitaker, 2021; Xiong et al., 2021), the role of online social media use to meet the social needs after the closure of physical social interactive places amid COVID-19 is critical (Haman, 2020; Jogezai et al., 2021; Yu et al., 2022). However, understanding the impact of COVID-19 on SME performance is critical. Then, our study is among the first to conduct a systematic survey on the impact of the COVID-19 pandemic on SMEs in a developing country, as many scholars only employed the qualitative survey or statistics descriptive to understand the impact of a pandemic. Overall, this research contributes to understanding those indicators most significantly affected by the pandemic. Indeed, the results of this study help quantify how the pandemic could generate different levels of impact on each indicator that could depend on the business and on what policymakers should consider as they contemplate the scale of the required intervention. They could then develop policy based on the impacted indicators, ensuring that policy measures are appropriately designed to meet the SME needs. Finally, by learning from the previous crisis, they could design an appropriate intervention to help SMEs survive, such as encouraging the SMEs to implement proactive sales to understand customer demand, building better customer and supplier relationships, and improving efficiency in resource utilization (Haluk Köksal and Özgül, 2007; Bourletidis and Triantafyllopoulos, 2014).

## Literature review

Recently, the negative impact of the COVID-19 pandemic on SME performance became a topic of interest for many researchers. In detail, Table 1 shows the country and object of the study, the method, and the negative impact of the pandemic on SME’s performance were retrieved from each study.

**TABLE 1:** Previous research on the impact of COVID-19 on SMEs.

No	Author (year)	Country	Object of study	Method	Impact
1	Abu Hatab et al., 2021	Egypt	166 agri-food	Survey with paper-based questionnaire; descriptive statistics and risk matrix (severity, likelihood, and immediacy of impact)	•Decreased in sales and revenue •Supply chain disruption (restrictions caused shipments grounded, transportation restriction and distribution disruptions, rejection of shipments, etc.) •Reduced labor productivity (employees are unable to commute to work, loss of skilled labor, reduced number of working days, increased work absenteeism) •Increased cost of production • Decreased financial assistance
2	Al-Fadly, 2020	Kuwait	25 SMEs in the hospitality and tourism sector (such as coffee shops, hotels, restaurants, and travel agents)	Unstructured interviews or observation; descriptive qualitative analysis	•Decreased the staff by between 20 and 50% with disrupted business Social distancing makes businesses like coffee shops and restaurants difficult to operates •Decreased orders from customers •Suppliers were unable to provide services and coordination between suppliers was difficult •Increased operating cost and financial burden •Decreased revenue
3	Bartik et al., 2020	United States of America	5,800 small businesses; not specific in certain sectors	Online-pulse surveys; descriptive statistics	•About 43% of businesses were temporarily closed and employment fell by 40% •Little cash on hand made SMEs either dramatically cut expenses, take on additional debt, or declare bankruptcy
4	Fabeil et al., 2020	Sabah, Malaysia	Two micro-entrepreneurs in the rural areas of Sabah	Phone interviews with open-ended questions; descriptive quantitative analysis	•Low product demand •Reduced income
5	Humphries et al., 2020	United States of America	More than 8,000 small business owners	Surveys—descriptive statistics	•About 60% had laid off at least one worker •Businesses reported an above 90% probability of permanent closure or bankruptcy within the next 6 months
6	Lu et al., 2020	Sichuan, China	4,807 SMEs	Questionnaire-based survey and semi-structured telephone interviews; descriptive statistics	•Unable to resume work because of a shortage of materials •Inability of employees to return to work •Disrupted supply chains, and reduced market demand •Cash flow risks because SMEs must pay for various fixed expenditures
7	Lutfi et al., 2020	Indonesia	587 SMEs	Observations, questionnaires, and literature studies; descriptive statistics	• Decreased income resulting from declining demand and problematic supply chains •Increased raw material costs and production costs
8	Mahajan, 2020	India	Not defined	Secondary data-descriptive qualitative analysis	•Decreased demand •Broken supply chain
9	Omar et al., 2020	Klang Valley and other states in Peninsular Malaysia	Six selected SMEs	Semi-structured telephone interview; descriptive qualitative analysis	• Operation disruption •Supply chain disruption •Problems in foreseeing business direction •Cash flow problems; risk of bankruptcy
10	Ratnasingam et al., 2020	Malaysia	748 SMEs in the furniture industry	Online surveys; descriptive statistics	•Raw material (uncertainty supply, unstable price, inconsistent quality, low stock in factory, long delivery time) •Employees (refusal to work for health concerns, absenteeism, low labor productivity, poor work attitude, poor quality) •Production (underutilized production capacity, low level of work in process, sub-standard quality, deteriorating workflow, deteriorating work environment) •Financial management (no cash flow, unable to service loans, unable to pay workers, unable to pay utilities, unable to pay creditors
					• Market demand (cancellation of export order, delay in shipment requested by customer, slow domestic market, slow repeat orders, poor payment cancellation) •Logistics (lack of transportation services, un-operational port services, high demurrage cost for shipment delays, lack of forwarding and shipping vessels, high freight insurance cost)
11	Shafi et al., 2020	Several cities in Pakistan	184 MSMEs	Surveys-online questionnaire; descriptive statistics	•About 31% reported shut down the business completely •About 43% reported lay off employees •About 12% reported reducing staff salary •About 44.83% reported supply chain disruption •About 44.02% reported decreased demand •About 38.04% reported decreased sales •About 41.85% reported decreased profit
12	Tairas, 2020	Several provinces in Indonesia	34 MSMEs (one in each province in Indonesia)	The interviews were carried out telephonically; secondary data was collected from websites of the statistical and news center agencies; descriptive qualitative analysis	•Difficulties in producing goods and services as a result of raw materials shortage •Decreased financial liquidity •Decreased demand
13	Nawaiseh, 2021	Jordan	1321 SMEs	Online questionnaire; descriptive statistics	•Operational difficulties (worker overload, low production, customer interests change, and low flow of raw material) •Financial challenges (low sales, cash flow, and turnover)
14	Saturwa et al., 2021	Several cities in Central Java Province, Indonesia	100 MSMEs	Online questionnaire; the paired *t*-test	•Decreased sales turnover •Decreased cash resilience
15	Dai et al., 2021	China	2,278 SMEs	Two-wave phone surveys; descriptive statistics; regression analysis	•Logistics blocks •Labor shortage •Reduced demand

According to Table 1, the impacts might vary according to the type of business activity. However, most authors concentrated on assessing the impact of COVID-19 on SMEs using descriptive statistics (Bartik et al., 2020; Humphries et al., 2020; Lu et al., 2020; Lutfi et al., 2020; Ratnasingam et al., 2020; Shafi et al., 2020; Abu Hatab et al., 2021; Dai et al., 2021). The negative impact of the COVID-19 pandemic on SMEs can be differentiated into six main categories: an employee-safe working environment (SWE), production process, markets and economic conditions, financial condition, public infrastructure, and political and regulatory environments.

(a) The pandemic has caused reduced labor productivity in an employee-SWE as employees cannot commute to work. It has also reduced the number of staff, reduced the number of working days, and increased work absenteeism as employees cannot return to work, or they refuse to work due to health concerns (Al-Fadly, 2020; Bartik et al., 2020; Humphries et al., 2020; Lu et al., 2020; Abu Hatab et al., 2021; Dai et al., 2021). In this case, employees are the heart of any organization, regardless of size or industry, which is why employee safety and well-being impact its short and long-term goals. As a result, the enterprise must maintain control over working conditions to provide a quality working environment for employees and increase a safe, positive atmosphere (Maamri et al., 2021).

Related to control over the working condition, according to Azizi et al. (2021), there are some practicing corporate social responsibility (CSR) in the context of human resource managers’ strategies for COVID-19 management, among others:

• Flexibility and employees’ virtual life cycle (such as flexibility of contracts, distance working, and working from home),
• Use innovative methods to support employees and maintain their health and welfare (such as helping and supporting employees by identifying where employees live; for an example, when employees have to cook for themselves),
• Use of staff safety measures and focusing on work conditions (such as the use of protective equipment, distribution of protective equipment to the employee at their discretion, observing of safety protocols by the employee, increasing the availability of testing health and safety of employees),
• Managers’ commitment (such as the obligation of administrative managers for supervision and monitoring during the working day in order to monitor the implementation of pandemic prevention measures, resolve disputes, obtain on-site feedback and take new measures),
• Selection and participation of the employee in decision-making (such as - creating an intimate atmosphere with employees and communicating with them by asking the question: (i) what factors cause your health to be good or bad here? (2) What factors can have a positive and not-so-positive effect on your health? (3) What factors can contribute to the health of the team/unit/employer?
• Making changes based on organization assessment and data (such as decision-making and continuous improvement of the safety, health, and well-being of employees by continuous monitoring carbon dioxide levels in indoor air, and the amount of fresh air in the workplace

Moreover, the company must meet the rights of its employees and society by preserving its workforce’s health and safety and improving working conditions (Ouffroukh et al., 2018). Occupational practitioners, healthcare advisers, and safety professionals should all be involved in developing adequate control measures, especially in the face of the new challenge of a pandemic situation (Maamri et al., 2021).

(b) In the production process, the pandemic has led to increased production costs, operational difficulties, permanent closure, operational disruption, underutilized or low production, deteriorating workflow and work environment (Al-Fadly, 2020; Humphries et al., 2020; Omar et al., 2020; Abu Hatab et al., 2021; Nawaiseh, 2021).

(c) In the market (on the demand side), the pandemic has led to decreased customer demand, sales, or orders, cancelation of export orders, and decreased sales turnover. However, on the supply side, the pandemic has led to difficulty in obtaining raw materials or services from suppliers; difficulty in supplier communications, increased cost of raw materials, logistical blocks, and supply chain disruptions (Al-Fadly, 2020; Fabeil et al., 2020; Lu et al., 2020; Lutfi et al., 2020; Mahajan, 2020; Omar et al., 2020; Ratnasingam et al., 2020; Shafi et al., 2020; Tairas, 2020; Abu Hatab et al., 2021; Dai et al., 2021; Saturwa et al., 2021). Supply-side is related to the entrepreneurial business network, a multifaceted network of business firms working together to achieve firm business objectives. (Eisenhardt and Martin, 2000; Ozcan and Eisenhardt, 2009). These objectives are typically operational and strategic, and business networks adapt them based on their role in the competitive environment in the market (Ford et al., 2014). There are two major categories of entrepreneurial business networks, namely business associations and business firm aggregations, which help SMEs become more dynamic, innovative, and competitive (Chung et al., 2015). An entrepreneurial business network is the socioeconomic business activity and a platform by which business executives and entrepreneurs meet to discuss available business network opportunities.

(d) Manufacturers are perpetually confronted with liquidity and profitability issues, and the COVID-19 pandemic has made them even more susceptible to economic shocks (Juergensen et al., 2020). During an economic storm, the manufacturing sector struggles with canceled orders, poor revenues, and falling stock prices (Handfield et al., 2020; Wuest et al., 2020; Tian et al., 2021). These market instabilities and unpredictability (Linton and Vakil, 2020; Paul and Chowdhury, 2020) cause panic in the industry, resulting in market anomalies and distorted supply-demand patterns (Khoo and Hock, 2020). So, to design an appropriate intervention, it is necessary to conduct a comprehensive assessment of the state of the businesses due to the new obstacles (Kapoor et al., 2021).

(e) In the financial condition of SMEs, the pandemic has led to decreased revenue, decreased profit, decreased financial liquidity, little cash on hand, reduced income, and cash flow risk or problems (Al-Fadly, 2020; Bartik et al., 2020; Fabeil et al., 2020; Lu et al., 2020; Omar et al., 2020; Ratnasingam et al., 2020; Shafi et al., 2020; Tairas, 2020; Nawaiseh, 2021). Generally, SMEs are financially fragile and smaller in size and resources; they are more vulnerable to the environmental crisis than their counterparts, i.e., large enterprises (Shafi et al., 2020). In this case, financial performance is an essential factor that may quantify economic pandemic effects on the companies’ function and existence. SMEs entrepreneurs more negatively perceived the financial performance of their companies during the pandemic as opposed to the period before the pandemic (Belas et al., 2021).

(f) In public infrastructure, the pandemic has led to a lack of transportation and un-operational services (Ratnasingam et al., 2020; Abu Hatab et al., 2021).

(g) In the political and regulatory environment, the pandemic has led to operational difficulties in businesses and the need for financial assistance (Al-Fadly, 2020; Abu Hatab et al., 2021).

## Method of research

### Study areas and sample of research

The object of research was SMEs in the Central Java Province. These SMEs were sampled to test the framework for measuring the impact of the COVID-19 pandemic on SME performance. There were 104 SMEs located in Semarang, Kudus, Jepara, Pati, Pekalongan, Magelang, Surakarta, Solo, and Cilacap that were sampled. Among the 104 sampled SMEs, 62.50% were in the furniture sector, 9.2% in the culinary sector, 2.88% in the handicraft sector, and 25% in the garment sector. Most of the sampled SMEs (102 SMEs or 98.08%) have fewer than 100 employees, and only two SMEs (1.92%) have over 100 employees.

This research employs non-probability convenient sampling to select the SMEs. The SMEs were selected from a community of individuals who were easy to reach or meet, such as friends, coworkers, classmates, and a WhatsApp group. The selection criteria also included their willingness to participate as respondents; they have been in operation for at least a year and were still operating throughout the COVID-19 pandemic. Although there are issues of generalizability in using the non-probability sample, developing research goals is scientifically warranted. Therefore, any sample coverage compromise is outweighed by the cost savings, convenience, target participant, and speed of response.

This study also used non-probability purposive sampling to choose respondents who completed the validation and analytic hierarchy process (AHP) questionnaire. These respondents should have sufficient knowledge of the impact of the pandemic on SME performance. This research comprised seven respondents who were willing to participate as the panel of experts. Those seven respondents are representatives from the Office of Cooperatives, Small and Medium Enterprises, Central Java Province (General Administration, Section Head of SME Production, Section Head of SME Financing, Division, Head of SME Program, Section Head of SME Marketing, Analyst of SME Financial Strategy, and an MSME consultant.

### Variable and measurement items

Initially, this study identified 54 indicators for measuring the impact of COVID-19 on SME performance. These indicators were developed using previous research (related to the impact of the COVID-19 pandemic as perceived by SMEs) and the indicators from previous measurement frameworks issued by organizations, such as the International Trade Centre (ITC) (International Trade Centre, 2020), Enterprise Survey for Innovation and Entrepreneurship in China (ESIEC) (ESIEC, 2020), and the International Labour Organization (ILO) (International Labour Organization, 2020). Given the content validity results, the number of indicators used to assess the impact of COVID-19 on SME performance was reduced from 54 to 44. Accordingly, the validation questionnaire had to be completed by a panel of experts with knowledge of the indicators for measuring the COVID-19 impact on SME performance. The panel had to evaluate if indications were relevant for measuring a particular dimension, establishing the domain of interest, and determining field conditions. The Content Validity Index (CVI) value was then used to assess the indicators’ validity (Haas and Springer, 2020). The CVI value for each indication is calculated by dividing the number of experts who gave a rating of 3 (relevant) or 4 (very relevant) for that indicator by the total number of experts, that is, the proportion of experts who agreed on relevance. For example, an item with a CVI of 0.80 would be judged as “relevant” or “very relevant” by four out of five assessors (Lynn, 1986). Haas and Springer (2020) established widely referenced standards for what constitutes an appropriate CVI value regarding the number of experts. When there are five or fewer experts, they believe the CVI should be 1.00, meaning that all experts must agree that the indicator is valid.

**FIGURE 1:**
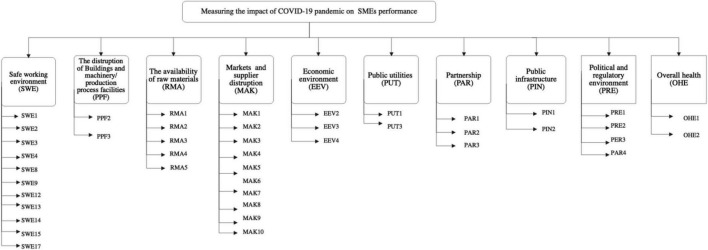
Hierarchical structure of indicators for measuring the impact of COVID-19 pandemic on SMEs performance.

Table 2 shows the results of the CVI computation for each indicator. Only indicators with a CVI of 0.867 or above were kept in this study. As seven experts were involved in the validation process, a CVI of 0.867 or above shows that the indicator is valid if no more than one expert disagreed with the indicator’s relevance. The following indicators were removed from the listed indicators for measuring the impact of the COVID-19 pandemic on SME performance: SWE5, SWE6, SWE7, SWE10, SWE11, SWE16, PPF1, EEM4, PUI2, and PAR4. So, after the content validation process, 44 indicators were used to measure the impact of the COVID-19. Then, the hierarchical structure of indicators for measuring the impact of the COVID-19 pandemic on SME performance can be seen in Figure 1 below.

**TABLE 2:** The results of the content validity of the indicators for measuring the impact of COVID-19 on SME performance.

Dimensions	No	Indicators	The number of experts who gave a rating of 3 or 4	CVI value
Safe working environment (SWE)	1	The number of COVID-19 cases in the geographical area of the enterprise operations (SWE1)	7	1,000
	2	The percentage of physically unsafe workers commuting to and from the workplace (e.g., using shared public transport, etc.) (SWE2)	6	0,857
	3	The increase of workers on sick/leave/absenteeism since March 2020 (SWE3)	7	1,000
	4	The possibility to rearrange work so workers can work from home (SWE4)	7	1,000
	5	The difficulty of sourcing adequate sanitation facilities (sanitizers, washing facilities, gloves, hand gels, masks, etc.) (SWE5) *	2	0,286
	6	The level of appropriateness of vehicles used for your business (e.g., vehicles for staff movement, delivery) with sanitizers and processes for regular cleaning (SWE6) *	5	0,714
	7	The percentage of workers who have responsibilities to take care of their family because of sick family members or school closure (SWE7) *	5	0,714
	8	The number of cases of internal transmission of COVID-19 by staff members or their immediate family members (SWE8)	7	1,000
	9	The percentage of workers feeling stressed with the working environment because of measures taken to address COVID-19 (SWE9)	7	1,000
	10	The percentage of workers who quit their job due to safety issues or another incident (SWE10)*	4	0,571
	11	The percentage of workers having close physical contact with customers/suppliers (SWE11) *	5	0,714
	12	Percentage of workers who have experienced personal trauma because a family member died or was sick due to COVID-19 (SWE12)	6	0,857
	13	Percentage of workers having to work close to the workplace for production/service delivery (SWE13)	6	0,857
	14	Number of staff members who have responsibility for giving recommendations related to COVID-19 as well as giving a daily review of official advice on risks on COVID-19 (SWE14)	6	0,857
	15	Existence of procedures for conducting self-checks to identify hazards that could lead to the spread of COVID-19 (e.g., conducting regular health and safety checks) (SWE15)	6	0,857
	16	The percentage of workers who have received training (or access to training) on COVID-19 preparedness and basic steps to protect themselves and others (SWE16) *	5	0,714
	17	Availability of a process to report to public health authorities any incident by workers or members of the public known or suspected to be related to the spread of COVID-19 (SWE17)	7	1,000
The disruption of Buildings and machinery/production process facilities (PPF)	18	The level of difficulty to get the required machinery and equipment from the supplier (PPF1) *	1	0,143
	19	The level of disruption or delay from the support services that the enterprise needs to maintain major equipment and machinery (PPF2)	6	0,857
	20	The level of insurance coverage of your business (e.g., equipment and livestock, workers) (PPF3)	6	0,857
The availability of raw materials (RMA)	21	The percentage of imported raw materials (RMA1)	6	0,857
	22	The length of delay you experience to acquires raw materials/required production input (RMA2)	7	1,000
	23	The level of difficulties for securing your raw materials and main stocks (RMA3)	6	0,857
	24	The degree of impact of increased government restrictive policies (e.g., increased health screening) on delays in shipping the product needed to your enterprise (RMA4)	7	1,000
	25	The number of locations of raw materials and/main stocks (RMA5)	6	0,857
Markets and supplier disruption (MAK)	26	The level of the negative impact of disruptions caused by the COVID-19 pandemic on your customers and their capability to purchase your products or services (MAK1)	7	1,000
	27	The level of the negative impact of official government measures relating to health concerns for the overall population on your business sales (MAK2)	7	1,000
	28	Percentage of goods or services intended to serve the non-domestic market (MAK3)	6	0,857
	29	The percentage of the market you serve is in a middle- to high-risk country (MAK4)	6	0,857
	30	The percentage of your sales has decreased in a middle- to high-risk country (MAK5)	7	1,000
	31	The level of disruptions experienced by your supplies because the government increase the restrictions policy (MAK6)	6	0,857
	32	The level of disruptions experienced by your suppliers that cause them to reduce their capability to supply to your enterprise (MAK7)	7	1,000
	33	The number of supply routes to contact the main suppliers (MAK8)	7	1,000
	34	The number of substitute suppliers that could deliver goods and services in the situation of disruption (MAK9)	7	1,000
	35	The level of dependency on foreign suppliers for most of the raw material and the main inputs required for your business (MAK10)	6	0,857
Economic environment (EEV)	36	The level of economic activity impacted by COVID-19, which directly affects your business and the market in which you operate, or you expect to operate (EEV1)	7	1,000
	37	The increase in unemployment rates in the market in which you operate (EEV2)	7	1,000
	38	The increased actual criminal activity or risk of a criminal activity that affects your enterprise as a consequence of the depressed economic activity (EEV3)	7	1,000
	39	The level of the negative impact of unexpected raised at the price of inputs and other goods needed for your business operations (EEV4) *	5	0,714
Public utilities (PUT)	40	The level of the negative impact of disturbances of main public utilities (electricity, water, telecoms, sanitation, and health) on the enterprise or on the market in which you operate (PUT1)	6	0,857
	41	The level of negative impact of disturbances of main public utilities on the staff of the enterprise (i.e., facilities of sanitation at their home) (PUT2) *	5	0,714
	42	The degree of increase of the costs related to public utilities (electricity, water, etc.) (PUT3)	6	0,595
Partnership (PAR)	43	The level of the negative impact of disruptions caused by COVID-19 on your competitors and their capability to continue competitive (PAR1)	7	1,000
	44	The probability of collaborating with competitors by sharing health and safety practices and equipment (PAR2)	7	1,000
	45	The probability of collaborating with competitors by sharing equipment (PAR3)	6	0,857
	46	The level of the negative impact of difficulty to access financial services providers (e.g., less choice of providers) on the enterprise (PAR4) *	4	0,571
Public infrastructure (PIN)	47	The level of the negative impact of limitations to access public infrastructure on the enterprise or the market in which you operate, or you expect to operate (PIN1)	7	1,000
	48	The level of the negative impact of raised costs of using main public infrastructure on the enterprise or on the market in which you operate (PIN2)	6	0,857
Political and regulatory environment (PRE)	49	The level of the negative impact of an unexpected change of rules (i.e., regulation and laws) on the enterprise or on the market in which you operate (PRE1)	7	1,000
	50	The level of the negative impact of raised uncertainty in regulations or policy on the enterprise or on the market in which you operate (PRE2)	6	0,857
	51	The level of the negative impact of an unexpected change of rules (i.e., regulation and laws) on the workers of the enterprise (PRE3)	6	0,857
	52	The level of the negative impact of no government subsidies on the enterprise and workers during the COVID-19 pandemic (PRE4)	6	0,857
Overall health (OHE)	53	The probability of “The State of Emergency” or limitations on freedom of movement put in place or threatened to be put in place (OHE1)	7	1,000
	54	Ownership of contingency plan for situations of crises (OHE2)	7	1,000

*Deleted from the list of indicators.

### Data collection procedure

Two sources of data, primary and secondary data, were used in this research. The primary data sources were the results of the completed web-based questionnaire and short telephone interviews with the SME owner and a representative from the Office of Cooperatives, Small and Medium Enterprises, Central Java Province. The purpose of the interview is to clarify the results of filling out the questionnaire by SMEs so that the respondents who fill out the questionnaire and those interviewed are the same SMEs. Then, the secondary data is intended to complete the documents (such as data related to sales, profit, etc.) that may be required based on the questionnaire results and interviews.

Three types of questionnaires were developed in this research. The first was a validation questionnaire with a 4-point Likert scale (1 = not relevant, 2 = slightly relevant, 3 = relevant, and 4 = very relevant) to assess the validity of the 54 indicators for measuring the impact of the COVID-19 pandemic on SME performance. Second, an AHP questionnaire with Saaty’s 9-point scale (Saaty, 1995) assessed the level of importance of each dimension and indicator. Third, a closed-ended questionnaire measured the current condition of each indicator. The third questionnaire also applied a 4-point Likert scale with different meanings, as detailed in Table 3. Both the second and third questionnaires only used valid indicators (44 indicators out of 54 indicators). All the questionnaires were web-based, developed, and produced using Google Forms. Google Forms is a cloud-based data management tool freely available on the Internet by Google Inc. It may be used and created by anybody using the Internet (Vasantha Raju and Harinarayana, 2016).

**TABLE 3:** Characteristics of each cluster.

Information		Cluster 1 resilient	Cluster 2 low vulnerability	Cluster 3 high vulnerability	Cluster 4 moderate vulnerability
Number of SMEs		11 SMEs (19.58%)	6 SMEs (5.71%)	35 SMEs (33.65%)	52 SMEs (52%)
Type of business	Furniture	45.45%	66.67%	68.57%	59.62%
	Garment	45.45%	0.00%	17.14%	21.15%
	Culinary	9.09%	16.67%	0.00%	13.46%
	Handicraft	0.00%	16.67%	14.29%	5.77%
Market	Domestic	81.82%	100.00%	77.14%	61.54%
	Export	18.18%	0.00%	11.43%	21.15%
	Domestic and export	0.00%	0.00%	11.43%	17.31%
Number of employees	Less than 15	81.82%	50.00%	14.29%	32.69%
	15 to 30	18.18%	16.67%	8.57%	15.38%
	more than 30	0.00%	16.67%	77.14%	51.92%

The seven URL copies of the first and second questionnaires were distributed to seven respondents through their emails, which consisted of the representative of the Office of Cooperatives, Small and Medium Enterprises, Central Java Province. In this case, the seven respondents should fill out a validation questionnaire (first questionnaire) by giving a value with a range of 1–4 on each of the indicators asked. Furthermore, approximately 2 weeks later, the seven respondents had to fill out the AHP questionnaire (second questionnaires) with a value of 1–9 to assess the importance level of each dimension and indicator. Then, the 125 URL copies of the third questionnaires were distributed to the SMEs through the owners’ emails or other social media accounts. In this case, the owners of SMEs should fill out the questionnaire by giving a value in the range of 1–4 according to the condition faced by the SMEs asked by each indicator. Although email distribution is thought to limit distribution to individuals with computers and email accounts, this was not the case in this study as the respondents’ profiles matched the survey requirements. There were also short telephone interviews with the representative of the Office of Cooperatives, Small and Medium Enterprises, Central Java Province, and the SME owners to further investigate the reasons for choosing a specific value or scale in the questionnaire.

**FIGURE 2:**
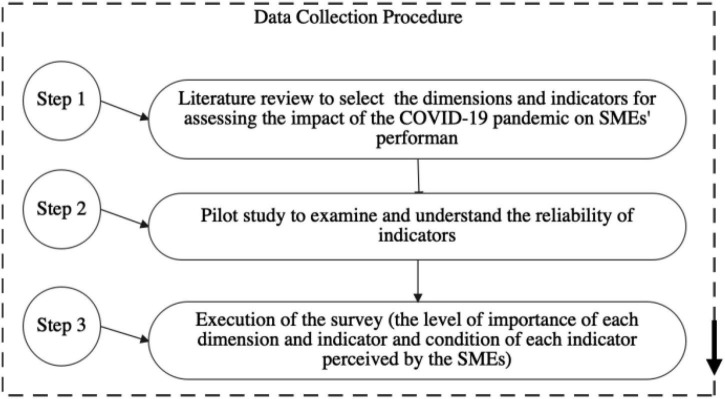
Data collection procedure.

Shortly, referring to Abbas et al. (2019), this study presents three phases of the data collection procedure. In the first step, this study selected the dimensions and indicators for assessing the impact of the COVID-19 pandemic on SMEs’ performance. In phase two, this study planned a pilot study to examine and understand the reliability of indicators and modified it accordingly if needed (first questionnaire or validation questionnaire). In the third and final phase, we conducted the survey and received data from respondents about the level of importance of each dimension and indicator (second questionnaire or AHP questionnaire). In the third phase, this study also conducted a survey and received data from respondents about the condition of each indicator perceived by the SMEs (third questionnaire). The three phases of the data collection procedure can be seen in Figure 2.

### Data processing procedure

Several different methods were used to analyze the data. The data from the first type of questionnaire were examined using content validity analysis (Lynn, 1986). The data from the second type of questionnaire were analyzed using AHP (Saaty, 1995). The data from the third type of questionnaire were examined using several approaches, such as simple descriptive statistics (statistical mean and simple graphics display), Multi-Attribute Value Theory (MAVT) approach (Keeney et al., 1993; Riabacke et al., 2012; Bottero et al., 2016), K-means cluster analysis (Xu and Tian, 2015), and discriminant analysis (Alayande and Adekunle, 2015; Keskin et al., 2020).

## Result and discussion

### Result of data processing with analytic hierarchy process

In this study, the AHP questionnaire evaluated the level of importance of each dimension and indicator to measure the impact of the pandemic on SME performance. Data were initially processed using AHP with a disseminated pairwise questionnaire given to the panel of experts, consisting of seven respondents or decision-makers. Each of the seven decision-makers initially completed the pairwise comparison at dimension and indicator levels separately. Then, the geometric mean of an individual’s judgments of the level of importance of each dimension and indicator was used to obtain a single value for each dimension and indicator. The geometric mean is used to find a combination of the answer of all decision-makers. In this case, the geometric mean was used to avoid a biased attitude of decision-makers toward a particular importance level (Dyer et al., 1992). Finally, each dimension’s global priority weight, the indicator’s local priority weight, and the consistency index (CI) were computed using the base of the combined or single value of pairwise comparisons data.

At level dimensions, based on the rearranged priority weight in descending order, the ranks of dimensions are as follows: SWE (0,2578), overall health (OHE) (0.2412), availability of raw material (RMA) (0.1147), market and supplier disruption (MAK) (0.1131), political and regulatory environment (PRE) (0.0965), partnership (PAR) (0.0582), the disruption of building and machinery production process facilities (PPF) (0.0476), economic environment (EEV) (0.1131), public utilities (PUT) (0.0184), and public infrastructure (PIN) (0.0965). In detail, each indicator’s local and global priority weight can be seen in the Supplemetary Appendix.

In the case of a SWE, research conducted by Zhou et al. (2021) suggests non-pharmaceutical interventions (NPIs) to combine various measures, such as the suppression strategy (lockdown and restrictions) and mitigation model, to decrease the burden on health systems. NPIs are significantly practical and helpful in reducing the quick transmission of the deadliest diseases. However, implementing the social distancing strategy is incredibly effective and beneficial, more so than other NPIs, to contain the rapid spread of the coronavirus. As a result, two or more synchronous NPIs are more productive and valuable than a single NPI strategy. In addition, the literature indicated that NPIs help significantly contains the rapid spread of the COVID-19 transmission. Based on the above debate, social distancing and implementing two or more NPI strategies can significantly contain the quick spread among people. Therefore, these strategies should be the priority in the ongoing panic situation of COVID-19.

### Result of data processing with multi-attribute value theory

Based on the local priority weight of each indicator and its measurement scale, the level of vulnerability of each SME during the pandemic can be assessed using the MAVT approach. Although MAVT includes different aggregation models, the simplest and most used one is the additive model (Belton and Stewart, 2002) as represented in the equation: V(a) = wi × vi(ai), where V(a) is the total value given to certain SMEs by considering all indicators simultaneously; wi is the weight assigned to reflect the importance of indicator i, and vi(ai) is the value reflecting certain SME performance on indicator i.

**FIGURE 3:**
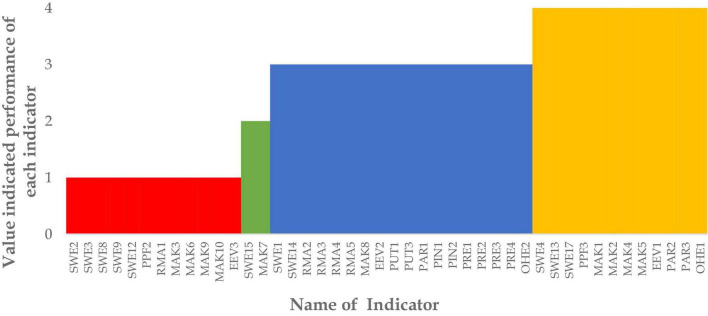
The mode value of each indicator.

As seen in Figure 3, only 13 of 44 indicators have a good performance. These indicators have a mode value of 1 or 2. However, most of the indicators proposed in this research have a mode value of 3 or 4. Accordingly, most of the proposed indicators have poor performance during the pandemic. There are 12 indicators with a mode value of 4 (the worst condition), namely SWE4, SWE13, PPF3, MAK1, MAK2, MAK4, MAK5, PAR1, PAR2, and OHE1.

**FIGURE 4:**
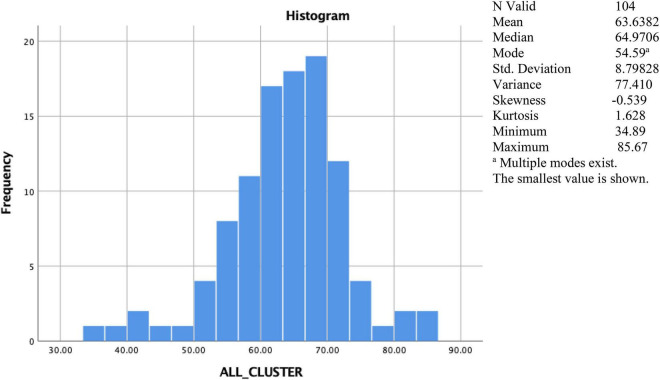
Histogram and the profile of total value of vulnerability given to each of the 104 SMEs.

This study was only able to collect the results of filling out questionnaires from 104 SMEs from 125 SMEs or a response rate of 82.3%. So, based on this condition, the histogram and descriptive statistics of the total value given to each of the 104 SMEs, by considering all indicators simultaneously, can be seen in Figure 4. This total value indicates the level of vulnerability of SMEs during the COVID-19 pandemic. The maximum number of vulnerability values achieved by each of the 104 SMEs is 100, as we converted marks from 4 to 100. We divided the total value achieved by four and then multiplied it by 100. Regarding the scale used by each indicator: the higher the total value given to SMEs, the more vulnerable the SME during the COVID-19 pandemic.

The average value of the level of vulnerability is 63.6382, with a median of 64.9706, a mode of the smallest value) 54.59, and a standard deviation of 8.798. The value of the level of vulnerability has a positive kurtosis value, indicating a “heavy-tailed” distribution.

### Result of data processing with cluster analysis

The K-means clustering algorithm segmented the level of vulnerability of SMEs because of the impact of the COVID-19 pandemic on SME performance. The values reflecting the SME performance for each of the 44 indicators were used as inputs in this algorithm. Referring to the value of the silhouette index (SI), the optimal number of segments equals 4 (four), meaning that four clusters have the highest SI value among other selected numbers of clusters (this study tried a different number of clusters, i.e., 2, 3, …, 6.). Xiong and Li (2013), Moulavi et al. (2014), and Tomasini et al. (2016) have compared SI with a set of other internal measures and proven it to be one of the most effective and generally applicable measures for clustering validity evaluation. The name of each cluster accords with ILO terminology (International Labour Organization, 2020), that is, resilient, low vulnerability, moderate vulnerability, and high vulnerability. Resilient means the SME is on the right path toward becoming more resilient, but some factors will reduce the SME’s vulnerability. Low vulnerability means the SME has increased preparedness, although it remains vulnerable. Moderate vulnerability means the SME has increased preparedness, but it remains vulnerable. High vulnerability means the SME was severely affected, which may cause long-term disruption should the situation deteriorate.

**FIGURE 5:**
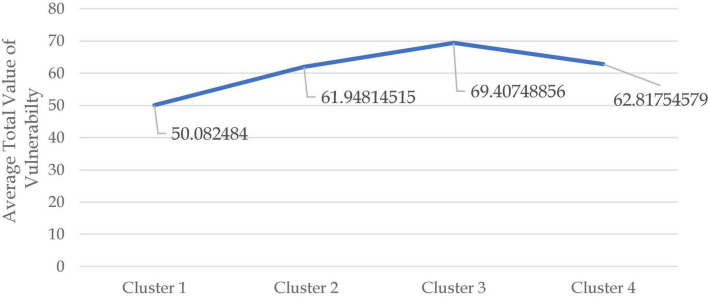
Histogram and the profile of total value of vulnerability given to each of the 104 SMEs.

**FIGURE 6:**
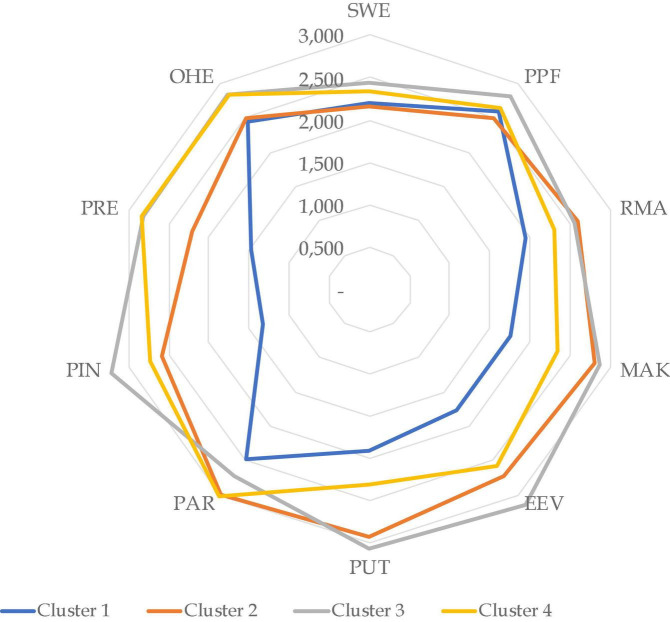
Radar charts according to the average value of the dimension for each cluster.

The average total vulnerability value of each cluster is shown in Figure 5 below. The SME profile in each cluster can be seen in Table 3, and the characteristics of the impact of the pandemic according to their dimensions in each cluster are shown in Table 4. Moreover, Figure 6 shows the radar chart comparing all segments according to the characteristics of the COVID-19 pandemic impact on each dimension. According to Figure 5, the average total value of vulnerability with the highest score is cluster 3 (69,4075), followed by cluster 4 (62.8176), cluster 2 (61,94815), and cluster 1 (50.0825). Thus cluster 1 is termed a resilient cluster. Cluster 2 is a low vulnerability cluster, and cluster 3 is a high vulnerability cluster. Cluster 4 is a moderate vulnerability cluster.

**TABLE 4:** The average value of the dimensions for each cluster.

Dimensions	Cluster 1 resilient	Cluster 2 low vulnerability	Cluster 3 high vulnerability	Cluster 4 moderate vulnerability
Safe working environment (SWE)	2,199	2,152	2,449	2,343
Buildings and machinery or production process facilities (PPF)	2,591	2,500	2,829	2,635
The Availability of Raw materials (RMA)	1,946	2,600	2,543	2,308
Markets and supplier disruption (MAK)	1,755	2,800	2,869	2,340
Economic environment (EEV)	1,758	2,722	3,143	2,583
Public utilities (PUT)	1,909	2,917	3,071	2,298
Partnership (PAR)	2,485	3,000	2,733	3,032
Public infrastructure (PIN)	1,318	2,583	3,214	2,731
Political and regulatory environment (PRE)	1,477	2,208	2,807	2,827
Overall health (OHE)	2,455	2,500	2,843	2,837

Referring to Table 3, cluster 1, or the resilient cluster, comprises 11 SMEs (10.58%) and SMEs in the furniture and garment sectors in a domestic market dominate. Most of the SMEs in cluster 1 have fewer than 15 employees. Cluster 2, or the low vulnerable cluster, comprises six SMEs (5.71%), SMEs in the furniture sector with a domestic market dominant. Like cluster 1, most SMEs in cluster 2 have fewer than 15 employees. Cluster 3, or the high vulnerability cluster, comprises 35 SMEs (33.65%), with domestic and overseas markets, where SMEs in the furniture sector dominate. Most of the SMEs in this cluster, about 77%, have over 30 employees. Cluster 4, or the moderate vulnerability cluster, comprises 52 SMEs (52%), where SMEs in the furniture sector dominate in domestic and overseas markets. The number of employees who worked in SMEs in cluster 4 varied significantly. About 32.69% of SMEs in cluster 4 have fewer than 15 employees, 15.38% of SMEs in cluster 4 have 15–30 employees, and 51.92% of SMEs in cluster 4 have more than 30 employees. As shown in Table 4 and Figure 6, cluster 3 has the highest impact in almost all dimensions of the clusters.

### Result of data processing with discriminant analysis

After conducting the clustering analysis to obtain the number of parts, the discriminant analysis was performed to determine any significant differences between the parts. The analysis also built a predictive model for each segment, group, or case membership through identification, using the best independent variables or indicators to distinguish between two or more segments, groups, or cases. These independent variables form a distinctive discriminant function (Alayande and Adekunle, 2015; Keskin et al., 2020).

In this study, the discriminant analysis started with calculating Wilk’s lambda to test the equality of the groups’ means for the same variables or indicators. Wilk’s lambda value varies from 0 to 1, where 0 indicates the differences in the parts’ means, and one indicates the similarity of all the segment means. The smaller the lambda value, the more the variable contributes to the discriminant function (Poulsen and French, 2004; Balogun et al., 2014, 2015). The Wilk’s lambda test statistic is designed to determine the discriminant function that maximizes the quotient between the variation explained by the difference between the segment means and the variation within these segments. As shown in Table 5 below, the smallest value of Wilk’s lambda is the MAK 7 indicator (the level of disruptions experienced by your suppliers that causes the supplier to reduce supply capability to your enterprise). It implies that this indicator is the one that provides the most significant difference between the means of the segments.

**TABLE 5:** Wilk’s Lambda test statistical results.

Indicators	Wilk’s Lambda	Indicators	Wilk’s Lambda	Indicators	Wilk’s Lambda	Indicators	Wilk’s Lambda
SWE1	0.9688	PPF2	0.8171	MAK5	0.8830	PAR1	0.9086
SWE2	0.8258	PPF3	0.9116	MAK6	0.5847	PAR2	0.8544
SWE3	0.9190	RMA1	0.5669	MAK7	0.5612	PAR3	0.9556
SWE4	0.9782	RMA2	0.8705	MAK8	0.8831	PIN1	0.7058
SWE8	0.9215	RMA3	0.9556	MAK9	0.8845	PIN2	0.7383
SWE9	0.8252	RMA4	0.8183	MAK10	0.6556	PRE1	0.7223
SWE12	0.8533	RMA5	0.9266	EEM1	0.6571	PRE2	0.7474
SWE13	0.9836	MAK1	0.8359	EEM2	0.7762	PRE3	0.8010
SWE14	0.8991	MAK2	0.6592	EEM3	0.8039	PRE4	0.8853
SWE15	0.8182	MAK3	0.9672	PUI1	0.6812	OHE1	0.9107
SWE17	0.9473	MAK4	0.9443	PUI3	0.8604	OHE2	0.9611

**FIGURE 7:**
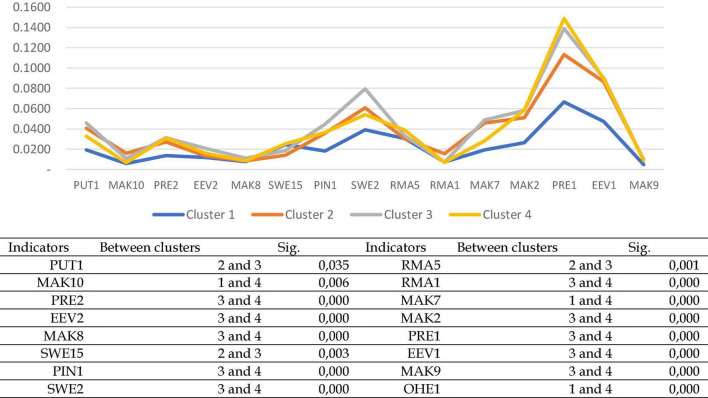
The result of checking the best indicators to identify four cluster segments in each function by entering or removing a variable.

The next step was to check the critical discriminating variables by entering a variable into the SPSS software or removing it from the SPSS software (see Figure 7 for the result). There were 16 indicators that could identify the differences between the four clusters. The 16 variables are PUT1, MAK10, OHE1, PRE2, EEV2, MAK8, SWE15, PIN1, RMA5, RMA1, MAK7, MAK2, PRE1, EEM1, and MAK9.

Based on the indicators to identify the four clusters, the Fisher’s linear discriminant function (FLDF) was arranged using SPSS software to predict the location of SMEs in a particular cluster. This function (FLDF) is frequently used for discrimination, classification, and prediction purposes under the usual basic statistical assumptions of multivariate normality of the independent variables, equality of variance and covariance matrices, and the relative equality of group sample sizes. Moreover, Fisher’s linear discriminant analysis technique was used to fit a predictive equation based on the measured variables for classifying new individuals and re-classifying the original data to interpret the predictive equation and better understand the relationships among the variables (Dibal and Abraham, 2020). The results of the FLDF from the SPSS software can be seen in Table 6.

**TABLE 6:** Fisher’s linear discriminant function (FLDF).

	Cluster 1 resilient	Cluster 2 low vulnerability	Cluster 3 high vulnerability	Cluster 4 moderate vulnerability
SWE2	85,2830	147,3580	167,3430	140,3180
SWE15	478,8140	330,4260	368,2010	532,7930
RMA1	1.622,9140	3.485,1350	1.448,8930	1.729,2910
MAK2	129,7920	293,2760	251,8950	261,3150
MAK7	(12,5210)	47,2330	154,3880	(34,1430)
MAK9	1.008,8780	1.972,0140	1.729,1670	1.700,6050
MAK10	771,5750	1.773,4200	1.322,6930	1.055,6910
EEM1	153,0460	289,8780	254,1350	281,3050
EEM2	178,5160	4,9780	471,0600	145,1580
PIN1	(1,2370)	(2,1210)	1,0510	(1,1350)
PRE1	5,0690	6,0070	6,1430	9,6540
PRE2	26,8980	56,2460	51,0330	50,0400
(Constant)	–28,8520	–89,1040	–73,6580	-64,0300

Based on Table 6, the equation for the level of vulnerability of cluster 1 is:


(1) $$\begin{array}{c} \mathrm{The}\mathrm{level}\mathrm{of}\mathrm{vulnerability}\mathrm{of}\mathrm{cluster1}\\ =-28,8520+\left(85,2830SWE2\right)+\left(478,8140SWE15\right)\\ +\left(1.622,9140RMA1\right)+\left(129,7920MAK2\right)\\ +\left(-12,5210MAK7\right)+\left(1.008,8780MAK9\right)\\ +\left(771,5750MAK10\right)+\left(153,0460EEM1\right)\\ +\left(178,5160EEM2\right)+\left(-1,2370PIN1\right)+\left(5,0690PRE1\right)\\ +\left(26,8980PRE2\right)\ldots \ldots \ldots . \end{array}$$

(2) Thelevelofvulnerabilityofcluster 2=-89,1040+(147,3580SWE2)+(330,4260SWE15)+(3.485,1350RMA1)+(293,2760MAK2)+(47,2330MAK7)+(1.972,0140MAK9)+(1.773,4200MAK10)+(289,8780EEM1)+(4,9780EEM2)+(-2,1210PIN1)+(6,0070PRE1)+(56,2460PRE2)……………

(3) Thelevelofvulnerabilityofcluster 3=-73,6580+(167,3430SWE2)+(368,2010SWE15)+(1.448,8930RMA1)+(251,8950MAK2)+(154,3880MAK7)+(1.729,1670MAK9)+(1.322,6930MAK10)+(254,1350EEM1)+(471,0600EEM2)+(1,0510PIN1)+(6,1430PRE1)+(51,0330PRE2)…………

(4) Thelevelofvulnerabilityofcluster 4=-64,0300+(140,3180SWE2)+(532,7930SWE15)+(1.729,2910RMA1)+(261,3150MAK2)+(-34,1430MAK7)+(1.700,6050MAK9)+(1.055,6910MAK10)+(281,3050EEM1)+(145,1580EEM2)+(-1,1350PIN1)+(9,6540PRE1)+(50,0400PRE2)………….

For example, the SME’s performance is as follows during the -19 pandemic:

• The percentage of physically unsafe workers commuting to and from the workplace is 0–10% (e.g., using shared public transport) (SWE2 = 4)
• Most of the areas already have procedures to conduct self-inspections to identify hazards that could lead to the spread of COVID-19 (e.g., conducting regular health and safety checks) (SWE15 = 2)
• The percentage of imported raw materials is less than 5% (RMA1 = 1)
• The negative impact of official government measures relating to health concerns for the overall population on your business sales caused sales of the product to decrease by more than 20% (MAK2 = 4)
• The level of disruptions experienced by suppliers caused 50% or more of the main suppliers to be unable to supply input to the enterprise (MAK7 = 4)
• The enterprise has two substitute suppliers that could deliver goods and services in case of disruption (MAK9 = 3)
• Less than 25% of critical inputs and raw materials need to come from foreign suppliers (MAK10 = 1)
• Effect of COVID-19 on economic activity has a high impact on the business or the markets you operate in, or you expect it to (EEV1 = 4)
• The unemployment rates in the markets in which you operate are 5% to 25% (EEV2 = 4)
• Restrictions to accessing public infrastructure have a significant impact on your business or the markets you operate in, or you expect it to (you have to close your operation) (PIN1 = 4)
• Increased costs of using key public infrastructure have a moderate impact on business or the markets you operate in, or you expect it to (you can operate at half of your capacity) (i.e., regulation and laws) on the enterprise or on the market in which you operate (PRE1 = 3)
• Increased uncertainty in the policy or regulatory environment has a significant impact on your business or the markets you operate, or you expect it to (you have to close your operation) (PRE2 = 4)

According to this information and equations 1–4, each cluster’s vulnerability level can be calculated. The calculation results indicated that the total value for the levels of vulnerability for cluster 1 to cluster 4 is 155.3039, 167.4348, 188.2879, and -48.7982, respectively. The calculation results indicated that the SMEs belonging to cluster 3 have high vulnerability as the value for the level of vulnerability for cluster 3 is higher than the others.

## Conclusion, theoretical implication, and practical implication

### Conclusion

This research conducted a quantitative analysis to answer the first three research questions. The results demonstrated how to design a framework to measure SME vulnerability in terms of its performance caused by the effects of the COVID-19 pandemic. The results also demonstrated how to segment the SMEs based on the performance of the indicators that were impacted by the COVID-19 pandemic. The measurement framework consisted of 44 indicators belonging to 10 dimensions. It was conducted using the MAVT approach, and the segmentation was conducted using the K-means clustering algorithm and discriminant analysis. The data on the impact of the pandemic on SME performance was collected through online web-based questionnaires completed by 104 SMEs. Those SMEs were located in several regions in the Central Java Province. The segmentation generated four clusters: resilient cluster, low vulnerability cluster, moderate vulnerability cluster, and high vulnerability cluster. Most of the surveyed SMEs fell into the moderate and high vulnerability clusters.

The differences between the clusters relied on 16 indicators. However, the differences between the low and moderate clusters relied on the ownership procedures for carrying out self-checks to identify hazards that could lead to the spread of the COVID-19 disease; the number of locations of suppliers or raw materials; and the level of impact of disruption of key public utilities on SME businesses. For comparison, most SMEs in the moderate vulnerability level already have procedures to conduct self-inspections. The market in which those SMEs operate was less affected by key public utility disruptions, and those SMEs have several suppliers in different locations. Consequently, if one of their suppliers cannot work because of policy restrictions or other operational reasons, the SMEs still have other suppliers to guarantee their production process. For example, the SMEs would have to use other suppliers who could deliver material if they could obtain material from their regular suppliers.

The differences between the moderate vulnerability cluster and the high vulnerability cluster rely on the levels of the impact of uncertainty in policy or regulatory processes; unexpected rule changes; official government measures relating to health concerns; limitation to public infrastructure access, and the level of impact the number of physically unsafe workers’ commuting to and from the workplace has on the SME business or on the market in which the SME operates. The differences between moderate and high vulnerability clusters also rely on the negative impact of unemployment on the SME’s market, the number of substitute suppliers, and the main supplier’s supply route. On average, SMEs in the high vulnerability cluster have a moderate impact because they decreased their sales by more than 20%. The raw material supply for production was disrupted as they used 25–50% less imported raw material. Indeed, the capability of SMEs and the market in which the SMEs operate to manage the sudden changes in government regulations and the ease of SMEs in getting raw materials for production became important factors that differentiated between being a resilient or a high vulnerability cluster.

### Theoretical implication and practical implication

This research contributes both to theory and practice. Theoretically, this research contributes to the literature on multi-criteria decision making, performance management, and the effects of the COVID-19 pandemic on SMEs in four ways. First, this research synthesized the findings of the reviewed studies of the impact of the pandemic on SME performance by grouping them into six main themes consisting of various SME-related issues, such as employee- SWEs, production processes, markets, and economic conditions, financial conditions, public infrastructure, and the political and regulatory environment. Second, this research synthesized the findings of the reviewed studies on the pandemic’s impact on SMEs by developing a framework based on multi-criteria decision-making for measuring the level of SME vulnerability caused by the effect of the pandemic on their operations. Third, this research found how the framework can help cluster SMEs into four main clusters: resilient, low vulnerability, moderate vulnerability, and high vulnerability. Accordingly, this research attempts to improve the understanding of the indicators in question: which indicators help differentiate the impact of the COVID-19 pandemic and provide a guide for future studies in this area. Fourth, in addition to clustering the SMEs, this research determined the equation to assess which cluster an SME can be placed in based on its indicator performance. Fifth, in addition to summarizing this research’s knowledge of the COVID-19 pandemic and SMEs, it outlined how this knowledge was acquired (methodology) and in which contexts the knowledge applies. These findings can help shape decisions about methodology and context in future work. Finally, this research identified gaps in the study domain of the impact of the COVID-19 pandemic on SME performance and suggested unique research questions and opportunities for meaningful future research to fill those gaps.

Practically, the results of this study make recommendations for SMEs and the government. Using a measurement framework and the discriminant equation that resulted from this research, SMEs can understand their level of vulnerability in terms of specific impacts of the COVID-19 pandemic. Thus, SMEs can adapt to the new situation quickly and reduce damage. However, one of the most important conclusions of the current research is that supplier and market disruptions can make SMEs more vulnerable. To reduce SME vulnerability during the COVID-19 pandemic, the SME management or owner should proactively and informatively communicate with their suppliers and customers.

The SMEs should also have a contingency plan to buy from numerous suppliers, changing from foreign to domestic suppliers. It is suggested that SMEs propose innovative marketing by adopting digital media and using the Internet for business operations. They should also manage customer loyalty as the pandemic has negatively affected business sales. By embracing digital media or the Internet, SMEs can present their products effectively despite customers being unable to visit their business premises. Consumers can have a thorough understanding of what products are available and which products they want. Consumers can communicate online with sellers about products without restraint during the pandemic. Furthermore, digital media enables users to display buyer testimonies, record the number of visitors, and make specific offers to consumers. These activities have proven effective in creating sales, attracting new customers, and maintaining SME performance.

Moreover, to manage customer loyalty, SMEs should offer more reasonable prices given high unemployment levels or other changes in regulations that could affect their markets. The SME should also consider designing alternatives for customers in restricted areas, such as providing collection and delivery services. The SME management or owner should maintain long-term relationships with customers, which are vital to maintaining sales. Strengthening customer relationships may help SMEs to maintain their performance.

It is recommended that the government consider the current situation and the level of SME vulnerability and reduce tax and other costs. Given their vulnerability, the government should offer SMEs a stimulus package to avoid enterprise closures and bankruptcies. The government should also assist SMEs in distributing their products or using SME services. The sudden changes in government regulations can affect SMEs or the markets they operate in or expect to operate. The most common type of assistance received by SMEs was the distribution of goods.

## Limitation and future research

Finally, we acknowledge some limitations of our study concerning the sample and employed techniques, which future research should address. First, this study was limited geographically. The sample in our study included a small number of cases, limited to only SMEs in several regions in the Central Java Province. Second, we only measured the impact of the COVID-19 pandemic on general SMEs and did not focus on particular sectors or industries. The inclusion of specific sectors or industries may identify a specific impact on how that sector or industry will be more severely affected than others. This limitation may also reveal an opportunity to compare resilient and vulnerable SMEs in different sectors or industries, thus generating a more reliable response. Future research could benefit from replicating our work in similar and dissimilar contexts: in each sector, business activity and size, and a wider geographical area could be considered, including a nationwide study. Such research could allow for more accurate measurements of the pandemic’s impact on each indicator’s performance based on sector or industry.

Future studies could adopt a complementary regression approach (OLS and PLS) and the ISM approach regarding the quantitative analysis techniques. In this case, the OLS and PLS estimations could determine the relationship between one or more explanatory variables (such as a SWE, the readiness of building and machinery or production process facilities, the availability of raw material), and the SME financial performance during the pandemic. Finally, the ISM approach could help us articulate the mental models of the relationship between the indicators used in this research.

## Data availability statement

The original contributions presented in this study are included in the article/Supplementary material, further inquiries can be directed to the corresponding author.

## Ethics statement

The study protocol was reviewed and approved by the Review Board of Industrial Engineering Department, Diponegoro University. Written informed consent from the participants was not required to participate in this study in accordance with the national legislation and the institutional requirements.

## Author contributions

AS, NP, and AB conceived and designed the study. FP participated in the acquisition of data. AS, NP, and FP analyzed the data, gave advice on methodology, and drafted the manuscript. AS and AB revised the manuscript. AS was the guarantor of this work and had full access to all the data in the study and takes responsibility for its integrity and the accuracy of the data analysis. All authors read and approved the final manuscript.
