# Supplementary material for: Assessing the impact of the COVID-19 pandemic on small and medium-sized enterprises performance
Source: Front Psychol. 2022 Oct 11;13:927628. doi: 10.3389/fpsyg.2022.927628 (PMC9595116; doi:10.3389/fpsyg.2022.927628)
Supplement: Supplementary file 1 [file Data_Sheet_1.docx]

# Appendix

Table X. The Local and Global Priority Weight of each Indicator and Measurement Scales

| **Dimensions (Weight)** | **Indicators** | **Local Priority Weight** | **Global Priority Weight** | **Measurement Scales** |
| --- | --- | --- | --- | --- |
| Safe working environment (SWE) (0.2578) | SWE1 | 0,2366 | 0,0610 | 1 = the geographical area of your operations included in the green zone (areas with no confirmed cases); 2 = the geographical area of your operations included in the yellow zone (areas with few cases of local transmission, but without clusters of community transmission); 3= the geographical area of your operations included in the red zone (areas with Covid-19 cases in one or more clusters with a high increase in cases); 4= the geographical area of your operations included in the black zone (areas with Covid-19 more than 2,000 cases) |
|  | SWE2 | 0,1284 | 0,0331 | 1 = 0–10% since March 2020; 2 = 11–20%; 3 = 21–30%; 4 = More than 30% |
|  | SWE3 | 0,1251 | 0,0323 | 1 = 0–10% since March 2020; 2 = 11–20%; 3 = 21–30%; 4 = More than 30% |
|  | SWE4 | 0,0637 | 0,0164 | 1 = very easy –more than 20% can work from home; 2 = 11–20%% can work from home; 3 = Only 10% can work from home; 4 = Difficult—cannot work from home |
|  | SWE8 | 0,2276 | 0,0587 | 1 = no cases since March 2020; 2 = between 1 and 5 cases; 3 = between 6 and 10 cases; 4 = more than 10 cases |
|  | SWE9 | 0,0165 | 0,0043 | 1 = no worker feeling stressful since March 2020; 2 = 1–5%; 3 = 6–10%; 4 = More than 10% |
|  | SWE12 | 0,0732 | 0,0189 | 1 = 0–10% since March 2020; 2 = 11–20%; 3 = 21–30%; 4 = More than 30% |
|  | SWE13 | 0,0601 | 0,0155 | 1 = 0–10%; 2 = 11–20%; 3 = 21–30%; 4 = more than 30% |
|  | SWE14 | 0,0141 | 0,0036 | 1 = more than 4 staff members; 2 = 3–4 staff members; 3 = 1–2 staff members; 4 = None |
|  | SWE15 | 0,0329 | 0,0085 | 1 = all of the areas have procedures to conduct self-inspections; 2 = most of the areas already have procedures to conduct self-inspections; 3 = only for certain areas of production; 4 = none |
|  | SWE17 | 0,0219 | 0,0056 | 1 = the process for reporting to public health authorities is available and very easy to access by the enterprises; 2 = the process for reporting to public health authorities is available but very difficult to access by the enterprises; 3 = all the areas have procedures to conduct self-inspections; 4 = none |
| Buildings and machinery/production process facilities (PPF) (0.0476) | PPF2 | 0,7800 | 0,03713 | 1 = no delay at all; 2 = one day to less than one week; 3 = one week to less than one month; 4 = more than one month |
|  | PPF3 | 0,2200 | 0,01047 | 1 = fully insured; 2 = 50% of business (e.g., workers, equipment, and livestock) has been insured; 3 = 50% of business (e.g., workers, equipment, and livestock) has been insured; 4 = none |
| The availability of raw materials (RMA) (0.1157) | RMA1 | 0,0575 | 0,0067 | 1 = less than 5%; 2 = 5% to less than 25%; 3 = 25% until less than 50%; 4 = more than 50% |
|  | RMA2 | 0,2838 | 0,0328 | 1 = no delay at all; 2 = one day to less than one week; 3 = one week to less than one month; 4 = more than one month |
|  | RMA3 | 0,3369 | 0,0390 | 1 = very easy; 2 = easy; 3 = standard (as usual); 4 = difficult |
|  | RMA4 | 0,2106 | 0,0244 | 1 = no impact at all; 2 = low negative impact; 3 = moderate negative impact; 4 = high negative impact |
|  | RMA5 | 0,1111 | 0,0129 | 1 = more than 5 locations; 2 = 4 or 5 locations; 3 = 2 or 3 locations; 4 = only one location; |
| Markets and Supplier Disruption (MAK) (0.1131) | MAK1 | 0,1915 | 0,0217 | 1 = not impacted at all; 2 = sales of your product decrease less than 10%; 3 = decrease 10% to 20%; 4 = decrease by more than 20% |
|  | MAK2 | 0,1503 | 0,0170 | 1 = not impacted at all; 2 = sales of your product decrease less than 10%; 3 = decrease 10% to 20%; 4 = decrease by more than 20% |
|  | MAK3 | 0,0319 | 0,0036 | 1 = less than 5%; 2 = 5% to less than 25%; 3 = 25% to less than 50; 4 = 50% or more |
|  | MAK4 | 0,2144 | 0,0242 | 1 = less than 5%; 2 = 5% to less than 25%; 3 = 25% to less than 50; 4 = 50% or more |
|  | MAK5 | 0,0209 | 0,0024 | 1 = less than 5%; 2 = 5% to less than 25%; 3 = 25% to less than 50; 4 = 50% or more |
|  | MAK6 | 0,1369 | 0,0155 | 1 = less than 5%; 2 = 5% to less than 25%; 3 = 25% to less than 50; 4 = 50% or more |
|  | MAK7 | 0,1349 | 0,0153 | 1 = less than 5% of main suppliers cannot supply input to the enterprise; 2 = 5% to less than 25%; 3 = 25% to less than 50%; 4 = 50% or more |
|  | MAK8 | 0,0323 | 0,0037 | 1 = have more than three supply routes to access the key supplier; 2 = have three supply routes to access the key supplier; 3 = have two supply routes to access the key supplier; 4 = only have one supply route to access the key supplier; |
|  | MAK9 | 0,0335 | 0,0038 | 1 = have more than three suppliers; 2 = have three suppliers; 3 = have two suppliers; 4 = only have one supplier; |
|  | MAK10 | 0,0534 | 0,0060 | 1 = less than 25% key inputs and raw materials needed came from foreign supplier; 2 = 25% to less than 50% key inputs and raw materials needed came from foreign supplier; 3 = 50% to less than 75% key inputs and raw materials needed came from foreign supplier; 4 = 75% or more key inputs and raw materials needed came from foreign supplier |
| Economic environment (EEV) (0.0353) | EEV1 | 0,7343 | 0,0259 | 1 = does not impact at all; 2 = impact of COVID-19 on economic activity has a lower impact on the business or the markets you operate in, or you expect it to; 3 = impact of COVID-19 on economic activity has a moderate impact on the business or the markets you operate in, or you expect it to; 4 = impact of COVID-19 on economic activity has a high impact on the business or the markets you operate in, or you expect it to |
|  | EEV2 | 0,1763 | 0,0062 | 1 = less than 5% since March 2020; 2 = 5% until less than 25%; 3 = 25% to less than 50; 4 = 50% or more |
|  | EEV3 | 0,0895 | 0,0032 | 1 = less than 5% since March 2020; 2 = 5% to less than 25%; 3 = 2 5% to less than 50%; 4 = 50% or more |
| Public utilities (PUT) (0.0184) | PUT1 | 0,8306 | 0,0153 | 1 = does not impact at all; 2 = disruptions of key public utilities (water, electricity, telecoms, health and sanitation) have less impact on your business or the markets you operate or you expect it to (you can operate at less than 75% of your capacity); 3 = disruptions of key public utilities (water, electricity, telecoms, health and sanitation) have a moderate impact on your business or the markets you operate or you expect it to (you can operate at half of your capacity); 4 = disruptions of key public utilities (water, electricity, telecoms, health and sanitation) have a significant impact on your business or the markets you operate in, or you expect it to (you have to close your operation) |
|  | PUT3 | 0,1694 | 0,0031 | 1 = decrease by less than 10%; 2 = same as last year; 3 = increase, but less than or equal to 10%; 4 = increase by more than 10% |
| Partnership (PAR) (0.0582) | PAR1 | 0,7182 | 0,04179924 | 1 = more than 50% of your competitors are impacted by COVID-19; 2 = more than 25% to 50% of your competitors are impacted by COVID-19; 3 = 25% or less of your competitors are impacted by COVID-19; 4 = none of your competitors are impacted by COVID-19; |
|  | PAR2 | 0,1217 | 0,00708294 | 1 = can collaborate with more than 50% of competitors in sharing health and safety practices or equipment; 2 = can collaborate with more than 25% to 50% in sharing health and safety practices or equipment; 3 = can collaborate with 25% of competitors in sharing health and safety practices or equipment; 4 = cannot collaborate with a competitor in sharing health and safety practices or equipment |
|  | PAR3 | 0,1601 | 0,00931782 | 1 = can collaborate with more than 50% of competitors in sharing equipment; 2 = can collaborate with more than 25% to 50% of competitors in sharing equipment; 3 = can collaborate with 25% of competitors in sharing equipment; 4 = cannot collaborate with a competitor in sharing equipment |
| Public infrastructure (PIN) (0.0161) | PIN1 | 0,8282 | 0,0133 | 1 = does not impact at all; 2 = restrictions to accessing public infrastructure have less impact on your business or the markets you operate (you can operate at less than 75% of your capacity); 3 = restrictions to accessing public infrastructure have a moderate impact on your business or the markets you operate (you can operate at half of your capacity); 4 = restrictions to accessing public infrastructure have a significant impact on your business or the markets you operate (you have to close your operation) |
|  | PIN2 | 0,1718 | 0,0028 | 1 = does not impact at all; 2 = Increased costs of using key public infrastructure have less impact on your business or the markets you operate in, or you expect it to (you can operate at less than 75% of your capacity); 3 = increased costs of using key public infrastructure have a moderate impact on your business or the markets you operate in or you expect it to (you can operate in half of your capacity); 4 = increased costs of using key public infrastructure have a significant impact on your business or the markets you operate in (you have to close your operation); |
| Political and regulatory environment (PRE) (0.0965) | PRE1 | 0,5417 | 0,0523 | 1 = does not impact at all; 2 = Sudden change of regulations has less impact on your business or the markets you operate or you expect it to (you can operate at less than 75% of your capacity); 3 = sudden change of regulations has a moderate impact on your business or the markets you operate or you expect it to (you can operate at half of your capacity); 4 = sudden change of regulations has a significant impact on your business or the markets you operate, or you expect it to (you have to close your operation) |
|  | PRE2 | 0,1127 | 0,0109 | 1 = does not impact at all; 2 = increased uncertainty in policy or regulatory environments has less impact on your business or the markets you operate or you expect it to (you can operate at less than 75% of your capacity); 3 = increased uncertainty in policy or regulatory environments has a moderate impact on your business or the markets you operate or you expect it to (you can operate at half of your capacity); 4 = increased uncertainty in policy or regulatory environments has a significant impact on your business or the markets you operate or you expect it to (you have to close your operation) |
|  | PRE3 | 0,2862 | 0,0276 | 1 = does not impact at all; 2 = sudden change of regulations has less impact on the workers (you can operate at less than 75% of your capacity); 3 = sudden change of regulations has a moderate impact on the workers (you can operate at half of your capacity); 4 = sudden change of regulations has a significant impact on the workers (you have to close your operation) |
|  | PRE4 | 0,0594 | 0,0057 | 1 = does not impact at all; 2 = the government by not yet introducing subsidies has less impact on your business and workers during Covid-19 (you can operate at less than 75% of your capacity); 3 = the government not yet introducing subsidies has a moderate impact on your business and workers during Covid-19 (you can operate at half of your capacity); 4 = the government by not yet introducing subsidies has a significant impact on your business and workers during Covid-19 (you have to close your operation) |
| Overall health (OHE) (0.2412) | OHE1 | 0,8089 | 0,1951 | 1 = does not impact at all; 2 = sudden change of regulations has less impact on your business or the markets you operate, or you expect it to (you can operate at less than 75% of your capacity); 3 = sudden change of regulations has moderate impact on your business or the markets you operate or you expect it to (you can operate at half of your capacity); 4 = sudden change of regulations has a significant impact on your business or the markets you operate or you expect it to (you have to close your operation) |
|  | OHE2 | 0,1911 | 0,0461 | 1 = does not impact at all; 2 = increased uncertainty in policy or regulatory environment has less impact on your business or the markets you operate or you expect it to (you can operate at less than 75% of your capacity); 3 = increased uncertainty in policy or regulatory environment has a moderate impact on your business or the markets you operate or you expect it to (you can operate at half of your capacity); 4 = increased uncertainty in policy or regulatory environment has significant impact on your business or the markets you operate or you expect it to (you have to close your operation) |
